# Exploratory Disposal and Reuse Feasibility Analysis of Winter Maintenance Wash Water

**DOI:** 10.1371/journal.pone.0149500

**Published:** 2016-02-23

**Authors:** Heather L. Ullinger, Marla J. Kennedy, William H. Schneider, Christopher M. Miller

**Affiliations:** Department of Civil Engineering, University of Akron, Akron, Ohio, United States of America; Purdue University, UNITED STATES

## Abstract

The Ohio Department of Transportation has more than 60 facilities without sewer access generating approximately 19 million gallons of winter maintenance wash water. Off-site disposal is costly, creating the need for sustainable management strategies. The objective of this study was to conduct an exploratory feasibility analysis to assess wash water disposal and potential reuse as brine. Based on a comprehensive literature review and relevant environmental chemistry, a sampling protocol consisting of 31 water quality constituents was utilized for monthly sampling at three geographically distinct Ohio Department of Transportation garages during the winter of 2012. Results were compared to local disposal and reuse guidance limits. Three constituents, including a maximum copper concentration of 858 ppb, exceeded disposal limits, and many constituents also failed to meet reuse limits. Some concentrations were orders of magnitude higher than reuse limits and suggest pre-treatment would be necessary if wash water were reused as brine. These water quality results, in conjunction with copper chemical equilibrium modeling, show pH and dissolved carbon both significantly impact the total dissolved copper concentration and should be measured to assess reuse potential. The sampling protocol and specific obstacles highlighted in this paper aid in the future development of sustainable wash water management strategies.

## Introduction

Heavy metals that are generated on the roadway are the result of brake dust, tire wear, road weathering, vehicle corrosion, exhaust emissions, and oil leaks. A study by Harrison et al. [1] found that brake dust accounts for 55.3% (±7.0%) of nonexhaust traffic particles. Due to its predominance, brake wear has been the focus of most research on traffic-related heavy metals. Garg et al. [2] calculated that brake wear alone accounts for 5.1–14.1 mg/mi of particulate emissions. Johansson et al. [3] reported that more than 90% of road traffic emissions of copper were due to brake wear, and as much as 50% of zinc emission may be due to brake wear.

Another source of traffic-related heavy metals is tire wear. Harrison et al. [1] found tire dust accounts for 10.7% (±2.3%) of nonexhaust traffic particles. Luhana et al. [4] found that specific tire-wear emissions vary considerably but estimate that passenger cars emit 64–579 mg/mi-vehicle. Many studies have concluded that zinc is the most abundant metal in tire wear [5,6]. Tire wear analysis by Budai et al. [7] cited annual zinc values from tire wear were 25,980 kg/year for urban traffic and 51,960 kg/year for highway traffic. The same study found these levels to be approximately 1,000 to 10,000 times higher than cadmium, copper, lead, or antimony.

While the previous studies focused on metal emissions, a study by Jang et al. [8] characterized heavy metal build up on the roadway. Analysis of 200 street sweepings samples showed high concentrations of zinc (46.7 mg/kg), copper (10.7 mg/kg), and barium (10.5 mg/kg). A subsequent study by Jang et al. [9] assessed the feasibility of beneficial reuse or disposal of street sweepings and results were compared to applicable limits of soil clean up target levels established by the Florida Department of Environmental Protection. The study concluded that reuse through land application was feasible even though copper, arsenic, and lead were above target levels.

The traffic-related heavy metals that are emitted and then deposited on the roadway exist mostly in the particulate form. They become airborne, attach to vehicle parts, and travel with the vehicle until it is washed. The complexity of wash water and the presence of many constituents complicate the management process. In 2004, Virginia Transportation Research Council (VTRC) assessed water quality from on-site retention ponds that collect truck wash water and storm water at Virginia Department of Transportation (DOT) maintenance facilities. Samples collected from 45 retention ponds across nine Virginia DOT districts were analyzed for chloride, total suspended solids, and oil and grease [10]. Constituent concentrations in these samples were relatively low, but this may be attributed to dilution by storm water. The study did not measure metal concentrations.

A 2004 Indiana Department of Transportation (INDOT) report documented the results of truck wash water quality samples taken after an oil/water separator. The study measured chloride, total suspended solids, and oil and grease [11] and found average chloride ion concentrations in the truck wash water were higher than the maximum contaminant level (MCL) for chloride (250 ppm) set by the United States Environmental Protection Agency (USEPA) National Secondary Drinking Water Regulation [12]. While this study concluded wash water could be reused for salt brine after a treatment process utilizing an oil/water separator and settling tank, it did not assess heavy metals which could restrict reuse.

Like INDOT, the Ohio Department of Transportation (ODOT) investigated the potential to reuse wash water as brine. A Hull & Associates [13] report documents the collection of four water quality samples taken from the Henry County Garage: one from the oil/water separator effluent, one from each of the two on-site ponds, and one from the storm water catch basin. Samples were analyzed for heavy metals, fluoride, cyanide, phosphorus, oil and grease, pesticides and herbicides, and methylene blue active substances. Sampling results indicated aluminium, copper, iron, zinc, and oil and grease concentrations exceeded applicable limits in the sample collected from inside the wash bay, requiring the garage to dispose of the water rather than reuse.

Currently, many maintenance facilities without access to sanitary sewer that do not have on-site treatment systems must collect their wash water and pay for its disposal. Miller et al. [14] conducted a detailed review of winter maintenance facilities for ODOT and found that there are 60 facilities without sewer access generating approximately 19 million gallons of wash water. They also evaluated 18 potential management strategies for ODOT and performed detailed cost analyses for 6 of them including site-specific conditions that directly affect the cost of alternative management strategies.

The articles cited above indicate there are many challenges, and heavy metals are likely an important part of effectively managing winter maintenance truck wash water. These previous studies do not include thorough water quality data, including the presence and concentration of heavy metals in wash water originating from winter maintenance operations. Wash water quality results are necessary to evaluate disposal and reuse options for the numerous ODOT facilities lacking sewer access. The objective of this study was to implement comprehensive sampling to evaluate the quality of water generated from the washing of salt trucks at ODOT maintenance facilities and to conduct a feasibility analysis to assess disposal and determine the potential reuse of the wash water as brine.

## Materials and Methods

### Wash Water Sampling

#### Locations

To assess wash water quality, samples were collected by Ohio Department of Transportation (ODOT) personnel (i.e. special permission was not required as they voluntarily provided the samples and field studies did not involve endangered or protected species) at three ODOT locations once per month during winter 2012 (January, February, March, April).Locations were chosen based on geographical diversity and included Location S, Location H, and Location M. Characteristics of each location are shown in Table 1. Location S, which exhibits the highest traffic counts, snowfall, and population, was selected because of the urban nature of the service area. Location H is a rural location and was chosen because the wash water is diluted in an on-site pond and then designated for brine production, depending on wash water quality. Location M, which has the lowest traffic counts, snowfall, and population, presented a unique case in which wash water is treated before it is used as brine.

**Table 1:** ODOT facility characteristics for each sampling location [15].

Location	Geographic Coordinates	# of Trucks	DVMT	Roadways	Snowfall	Population
S	40.906738,-81.427632	4	15,507,060	95% Urban	High	332,082
H	41.405748,-84.051713	10	861,740	83% Rural	Medium	29,210
M	39.05967,-81.959871	15	577,490	83% Rural	Low	23,072

DVMT = Daily Vehicle Miles Travelled

#### Sampling and Analytical Procedures

The metal and nonmetal constituents selected for this study were chosen to adequately characterize and quantify the wash water and assess factors controlling the measured concentrations. The goal of sampling was to obtain wash water samples directly after treatment by an oil/water separator. Grab samples were taken during truck washing operations. At all three sites, samples were collected using a siphon pump from an access point after the oil/water separator (applies to samples S1, M1, and H1). Two additional sampling locations included a retention pond at Location H (sample H2) and a post-treatment sample at Location M (sample M2).

Each sample was placed in a 0.5 L high-density polyethylene bottle. A portion of the sample volume (125 mL) was preserved with (1+1) reagent grade nitric acid to pH < 2. The remaining sample volume was left unpreserved for nonmetals analysis. The preserved samples were digested according to EPA 200.7 and analyzed by Inductively Coupled Plasma—Optical Emission Spectrometry (ICP-OES).

Unpreserved samples were analyzed by the following methods: chloride (HACH 8113), conductivity (HACH CO150 Conductivity Meter), cyanide (HACH 8027), fluoride (HACH 8029), herbicides (EPA SW-846 Method 8151A), oil and grease (EPA Method 1664A), pesticides (EPA Method 608), pH (Fisher Scientific AB15 pH Meter), phosphorus (HACH 8048), total dissolved solids (TDS) (Standard Method 2540C [16]), dissolved organic carbon (DOC) (Shimadzu TOC-5000A TOC Analyzer), total suspended solids (TSS) (Standard Method 2540D [16]), turbidity (HACH 2100Q Portable Turbidimeter), and surfactants (HACH 8028).

### Disposal-Reuse Feasibility Analysis

#### Disposal and Reuse Limits

Two possible disposal methods for wash water are discharge to a local sanitary sewer system or transportation to a local wastewater treatment plant. Disposal limits in both cases are regionally dependent. For this study, a wastewater treatment plant in Ashtabula County, Ohio, provided suggested disposal limits. Reuse limits are regionally dependent as well. In some cases, reused water is not required to meet any water quality standard; in the other cases, water must meet local aquatic, agriculture, and wildlife standards. For this discussion, the phrase “reuse limit” is meant to summarize these aquatic, agriculture, and wildlife standards. In general, reuse limits are much more stringent than disposal limits.

Reuse limits for Location H have been determined based on a prior permit and are assessed using the most stringent criteria found in the following sources: Lake Erie Basin Aquatic Life Outside the Mixing Zone Maximum (OMZM) from Chapter 3745–1 of the Ohio Administrative Code (OAC); Statewide Water Quality Criteria for the Protection of Agricultural Uses (Table 7–12 of the State of Ohio Water Quality Standards Chapter 3745–1 of the OAC, with a 3.11 times conversion to maximum concentration); Statewide Water Quality Criteria for the Protection of Aquatic Life for Water Hardness Dependent Criteria (Table 7–9 of the State of Ohio Water Quality Standards Chapter 3745–1 of the OAC, using OMZM and hardness 288 mg/L); and Lake Erie drainage basin water quality criteria for the protection of human health and wildlife (Table 33–2 of the State of Ohio Water Quality Standards Chapter 3745-1-33 of the OAC, using Outside the Mixing Zone Average [OMZA]). All references to the OAC originate from Ohio Environmental Protection Agency [17].

Currently, there are no known reuse limits required for Location S. If this were to change, the projected reuse limits for Location S would be based on the same standards as Location H. The reuse limits for Location M are based on the following standards: Ohio River Basin Aquatic Life OMZM from Chapter 3745–1 of the OAC; Statewide Water Quality Criteria for the Protection of Agricultural Uses (Table 7–12 of the State of Ohio Water Quality Standards Chapter 3745–1 of the OAC, with a 3.11 times conversion to maximum concentration); Statewide Water Quality Criteria for the Protection of Aquatic Life for Water Hardness Dependent Criteria (Table 7–9 of the State of Ohio Water Quality Standards Chapter 3745–1 of the OAC, using OMZM and hardness dependent); and Lake Erie drainage basin water quality criteria for the protection of human health and wildlife (Table 33–2 of the State of Ohio Water Quality Standards Chapter 3745-1-33 of the OAC, using OMZA). While Table 33–2 is a standard for the Lake Erie drainage basin, the OMZA criteria for human health and wildlife are required to be maintained for the entire state.

#### Chemical Equilibrium Modeling

The importance of analyzing a comprehensive set of constituents lies in the complexity of the wash water. Heavy metals in wash water exist in several forms and interact with organics and inorganics present in the aqueous environment. Eq 1 shows that total metal concentration is comprised of solid and dissolved metals. The dissolved concentration is composed of the free metal ion, inorganic-complexed metals, and organic-complexed metals, as shown in Eq 2.

$${\left[Me\right]}_{total}={\left[Me\right]}_{solid}+{\left[Me\right]}_{dissolved}$$ (1)

$${\left[Me\right]}_{dissolved}={Me}^{+}+\sum_{i}[MeX_{i}]+\sum_{i}\left[MeL_{i}\right]$$ (2)

Where:

*Me* represents a metal

*Me*^+^ is the free ionic metal

*MeX*_*i*_ is the complex formed by the metal bound to an inorganic ligand

*MeL*_*i*_ is metal bound to an organic ligand

These equations show that, due to complexation, other constituents present in the wash water affect how the metal speciates, as well as the total dissolved metal concentration. Complexation affects solubility, toxicity, surface properties of solids, and adsorption ability of metals from the solution [18]. Understanding complexation and speciation is significant when analyzing management options or feasibility of reuse.

To better understand copper speciation, model chemical equilibrium calculations were performed with Visual MINTEQ v. 3.0. The Gibbs free energy was calculated for the formation of the solids CuCO3, CuO, Cu2O, and Cu(OH)2 under standard conditions and all free energy values and solubility product constants and equations were obtained from Visual Minteq. Metal binding by organic matter was modeled using the NICA-Donnan model and its default parameters. A solid copper concentration estimate of 10 mg/kg was used for the wash water particulates. To understand the effects of DOC and pH on metal speciation, general water quality parameters from USEPA [19] were entered into the model. These water quality measurements represent a typical municipal water source comparable to the water that is used to wash salt trucks in Ohio. Jang et al. [8] measured the total recoverable copper concentration in Florida, and this concentration is within the reported range of solid concentrations from street sweepings and catch basins.

## Results and Discussion

### Wash Water Sampling Results

Heavy metals in wash water originate from numerous traffic sources, including brake and tire wear, oil leaks, exhaust emissions, and vehicle and road corrosion. Each source contributes several metals to the environment. Table 2 shows that 9 of the 16 metals tested were present in 100% of the wash water samples (Group A), 2 metals were detected in 63% of samples (Group B), and 5 metals were detected in less than 16% of samples (Group C). The results of this sampling protocol confirm the presence of several heavy metals in truck wash water. This conclusion is supported by a comprehensive literature review, such as that by Luhana et al. [4], which indicates tire and brake wear alone can generate as many as 24 metals.

**Table 2:** Total concentrations of heavy metals at ODOT locations for samples taken from January–April 2012.

Group	Metal	Taken after Oil/Water Separator						Taken from Pond		Taken after Filtration	
		Location S1		Location H1		Location M1		Location H2		Location M2	
		Range (ppb)	Avg ± StdDev(ppb)	Range (ppb)	Avg ± StdDev(ppb)	Range (ppb)	Avg ± StdDev(ppb)	Range (ppb)	Avg ± StdDev(ppb)	Range (ppb)	Avg ± StdDev(ppb)
A	Zn	600–1245	884±267	63–243	153±92	100–1087	465±541	55–975	372±414	62–463	249±167
	Cu	275–799	555±282	9–387	125±176	563–858	666±166	10–378	144±164	24–815	451±415
	Fe	1026–3562	1805±1181	569–2420	1327±906	305–7195	3744±3445	45–12042	3525±5712	866–2442	1835±683
	Al	321–1196	584±411	101–524	265±194	34–688	411±339	60–6253	1898±2927	133–244	198±49
	Mn	281–456	347±76	141–229	183±41	300–869	630±295	2–342	103±160	631–1017	804±180
	B	55–111	85±23	76–161	115±36	40–48	45±4	26–100	50±34	40–78	51±18
	Ni	21–73	42±24	16–41	24±12	27–34	30±3	13–83	34±33	29–57	37±13
	Li	9–14	11±2	10–95	52±46	7–22	13±8	2–25	10±10	6–26	13±9
	Cr	8–19	13±5	2–8	4±3	1–3	2±1	1–11	5±4	2–4	3±1
B	Pb	52–188	92±64	28	28±0	41–45	43±3	25–160	93±95	25–64	39 ± 22
	Mo	5–12	8±4	2–14	8±5	2	2±0	6–11	8±2	--	--
C	V	3	3 ± 0	2	2±0	--	--	12	12±0	--	--
	Be	0.7	0.7 ± 0	1.7	1.7±0	--	--	--	--	--	--
	As	15	15 ± 0	--	--	--	--	--	--	--	--
	Cd, Co	--	--	--	--	--	--	--	--	--	--

Notes

--no detects

Location S1, Location H1, and Location M1 samples were taken directly after an oil/water separator.

Location H2 sample was taken from a pond and Location M2 sample was taken after a treatment unit.

Samples were taken once per month at each of the five locations. Monthly samples were taken from January 2012 to April 2012 for a total of 19 samples.

Luhana et al. [4] reported results comparable to samples taken at Location S1, H1, and M1. At these locations, samples were taken directly after the oil/water separator and should reflect contaminants present on the trucks. Table 2 shows that iron, zinc, and copper exhibit the highest average concentrations at these three locations. Luhana et al. [4] found that zinc is the most prevalent metal, and iron is the third most prevalent metal in tires; in brake linings, iron and copper are the most and second most prevalent metals, respectively. As noted by Westerlund [20], heavy metal content in brake linings can vary by several orders of magnitude due to manufacturer, type, and model of the brakes. Tire compositions have similar variations and usually are not disclosed for proprietary reasons [7].

General wash water quality measures are expected to impact the heavy metal concentration (Table 3). For example pH, with a notable range of 6.3–7.4, will influence metal solubility. In addition, the measured chloride concentrations range from 600–18,600 mg/L; this is a significant variation in a known metal ligand that could also affect the total dissolved metal concentration.

**Table 3:** Concentrations of nonmetal water quality constituents at ODOT locations for samples taken from January–April 2012.

Constituent	Taken after Oil/Water Separator						Taken from Pond		Taken after Filtration	
	Location S1		Location H1		Location M1		Location H2		Location M2	
	Range	Avg ± StdDev	Range	Avg ± StdDev	Range	Avg ± StdDev	Range	Avg ± StdDev	Range	Avg ± StdDev
Chloride (as Cl-)	1400–12300	7225±4545	600–18600	7575±8490	3300–5200	4167±961	500–6000	2425±2451	1400–4800	3600±1506
Conductivity (mS)	6–40	26±14	4–62	26±28	16–20	18±3	2–26	10±11	7–20	15±6
Cyanide (as CN-)	0.009–0.036	0.022±0.013	0.011–0.087	0.037±0.035	0.003–0.007	0.005±0.002	0.002–0.113	0.032±0.054	0.004–0.015	0.009±0.005
DOC	13–52	24±19	8–64	37±26	7–8	8±1	2–5	4±1	6–18	11±5
Fluoride	0.2–1.2	0.8±0.4	0.5–0.9	0.6±0.2	0.8–1.3	1.0±0.3	0.4–1.2	0.6±0.3	0.7–1.1	0.9±0.2
Herbicides (μg/L)	--	--	336	336±0	--	--	--	--	--	--
Oil and Grease	13–120	51±60	9–27	18±9	--	--	--	--	--	--
Pesticides (μg/L)	--	--	--	--	--	--	--	--	--	--
pH	6.4–6.9	6.6±0.2	6.3–6.8	6.7±0.2	6.8–7.0	6.9±0.1	6.6–7.4	6.9±0.4	6.8–7.0	6.9±0.1
Phosphorus (as PO43-)	0.01–0.12	0.09±0.05	0.02–0.31	0.16±0.14	0.14–0.77	0.38±0.34	0.01–0.26	0.14±0.12	0.17–1.32	0.51±0.54
Selenium	--	--	0.12	0.12±0	--	--	--	--	--	--
Surfactants	0.04–1.91	0.53±0.92	0.08–4.13	1.19±1.96	0.04–0.05	0.05±0.01	0.02–0.05	0.03±0.01	0.03–0.10	0.06±0.03
TDS	3510–24490	15973±8979	1950–45260	17268±20252	9120–11460	10520±1236	1340–15740	5888±6638	3670–11460	8713±3620
TSS	48–397	150±166	76–153	108±33	4–44	19±22	66–2092	777±1140	12–224	72±102
Turbidity (NTU)	47–245	108±92	28–212	102±80	2–91	47±44	<1–1569	415±770	9–26	18±8

Notes

--no detects

Units are mg/L unless otherwise noted.

Herbicide detect was for 2,4 –D.

DOC = Dissolved Organic Carbon, TDS = Total Dissolved Solids, TSS = Total Suspended Solids.

Location S1, Location H1, and Location M1 samples were taken directly after an oil/water separator.

Location H2 sample was taken from a pond and Location M2 sample was taken after a recycle unit.

Metal concentrations shown in Table 2 were also compared to wash water results from another ODOT study [13]. Copper, chromium, iron, and vanadium were exceptions, with concentrations orders of magnitude higher in the 2010 study. As described in the 2010 study, the sample was collected from the sump located in the wash bay prior to the oil/water separator. Lower metal concentrations found in samples from Location S1, H1, and M1 in this study show that treatment with an oil/water separator likely reduces metal concentrations. Elevated concentrations of oil and grease, surfactants, and TSS in wash water samples could be used to indicate less than optimal operation and maintenance of the oil/water separator.

While the location of the sample collection (i.e., whether the sample was collected before or after an oil/water separator) is important, it is also important to consider the location and environment of truck service areas. Traffic-related heavy metals, such as those discussed above, are more abundant in urban environments due to frequent deceleration, acceleration, and turning motions [7]. Results presented in Table 2 show that Location S1 has the highest average concentration for four of nine Group A metals. This result is expected given that Location S1 consists of 95% urban roadways, with nearly 18 times the traffic of Location H and 27 times the traffic of Location M.

### Feasibility Analysis

#### Comparison of Data to Disposal-Reuse Limits

Despite variation in the data, copper and zinc are consistently found in elevated concentrations. These elevated concentrations present challenges to managing wash water. Table 4 compares each sample with limits for disposal and reuse management options. When exceedances from Table 4 are tallied, Location S1 produces the highest number of metal exceedances; all are reuse exceedances for copper and zinc. Recall from Table 2 that copper and zinc had some of the highest concentrations, and that Location S1 had the highest concentrations of several metals due to the urban nature of the site. Location S1 also had the highest number of nonmetal exceedances: most notably, oil and grease. Overall, Table 4 shows that most samples meet limits for disposal to a sanitary sewer. Calculations based on this table show that only 4 of 19 samples exceed limits for disposal, while 17 of 19 samples exceed limits set for reuse. This makes disposal a much more likely option but indicates that treatment may be required before wash water is disposed.

**Table 4:** Concentrations of metal and nonmetal constituents that exceed disposal and reuse.

		S1		H1		H2		M1		M2	
Month	D or R	M	NM	M	NM	M	NM	M	NM	M	NM
Jan.	D	✓	TSS 397 O&G 120	✓	✓	✓	TSS 2092			✓	✓
Jan.	R	Cu 799 Zn 830	CN 0.036 O&G 120	Cu 387	Surf. 4.0	Cu 378 Zn 975	✓			Cu 165	✓
Feb.	D	✓	✓	✓	✓	✓	✓	✓	✓	✓	✓
Feb.	R	Cu 349 Zn 600	CN 0.029	✓	O&G 27	✓	✓	Cu 563	✓	✓	✓
Mar.	D	✓	✓	✓	✓	✓	✓	Cu 1658	✓	✓	✓
Mar.	R	Cu 797 Zn 1245	O&G 19	Cu 68	CN 0.087	Cu 55	✓	Cu 1658	✓	Cu 815	✓
Apr.	D	✓	✓	✓	✓	✓	✓	Cu 858	✓	✓	✓
Apr.	R	Cu 275 Zn 860	O&G 13 Surf.1.91	✓	CN 0.038 O&G 19	Cu 131 Zn 299	✓	Cu 858 Zn 1087	✓	Cu 800 Zn 463	✓
	Exc.	8	8	2	5	5	1	6	0	4	0

Notes

Metals are expressed in μg/L. Nonmetals are expressed in mg/L. No sample was taken from M1 in January.

‘✓ ‘ indicates that the sample met all limits.

Value following the name of the constituent is the concentration found in that particular sample.

CN = cyanide, Cu = copper, D = disposal, Exc. = number of constituents exceeding limits, M = metals, NM = non-metals, O&G = oil and grease, R = reuse, Surf. = surfactants, TSS = total suspended solids, Zn = zinc.

Additional treatment in the form of dilution occurs at Location H. The sample taken at Location H2 was collected from the on-site retention pond, which captures truck wash water and runoff from the entire maintenance yard. It is expected that diluted samples would have lower metal concentrations; however, Table 2 shows this is not the case. For example, lead and zinc exhibited concentrations more than 100% higher in the pond sample. Table 4 confirms dilution is not an adequate treatment for meeting limits. Samples from Location H2 exceed reuse limits for copper and zinc and disposal limits for TSS. One plausible explanation for Location H2’s exceedances is that the pond receives runoff water from the entire site, which creates a composite effect that may increase metal concentrations in the pond. Another possible explanation for Location H2’s exceedances is that pond sediments may have been stirred during sampling. Stirring causes high TSS levels and disturbs metals that previously settled into the sediment.

Additional treatment also occurs at Location M, which utilizes a clay-based filtration system. For this reason, Location M2 was expected to have lower concentrations of metals. However, when compared with Location M1, Location M2 only exhibited lower concentrations for four of nine Group A metals. Table 4 confirms this treatment system does not adequately remove heavy metals, as copper and zinc still exceed reuse limits.

The results discussed above indicate that even after wash water is treated by the oil/water separator, and sometimes additional treatments such as dilution and filtration, further treatment is required to meet reuse and disposal limits. Further treatment should focus on the constituents presented in Table 5, which summarizes exceedance results. Table 5 lists constituents that exceed limits for disposal and reuse management options and the percent of samples that exceeded the limit for each constituent.

**Table 5:** List of constituents that exceed disposal and reuse limits, followed by the percentage of samples exceeding the limits for that particular constituent.

Disposal		Reuse	
Constituent	%	Constituent	%
Copper	11%	Copper	79%
Oil and Grease	11%	Oil and Grease	26%
Total Suspended Solids	5%	Zinc	42%
		Cyanide	21%
		Surfactants	11%

As shown in Table 5, copper and oil and grease create challenges for both management options. Seventy-nine percent of samples do not meet the reuse limits for copper. Copper also exceeds disposal limits but at a lesser margin of 11%. In addition to copper, oil and grease fails to meet disposal limits in 11% of samples and reuse limits in 26% of the samples. Other constituents that exceed reuse standards are zinc (41%), cyanide (21%), and surfactants (11%). Most constituents exceed reuse limits by 100–1,000%; however, copper shows an even greater exceedance of as much as 4,151%. The results of this analysis show that both management options require pretreatment. Pretreatment should focus on the constituents listed in Table 5, taking into account the large percentage by which these concentrations exceed limits.

#### Copper Chemical Equilibrium Modeling and Analysis

**Fig 1:**
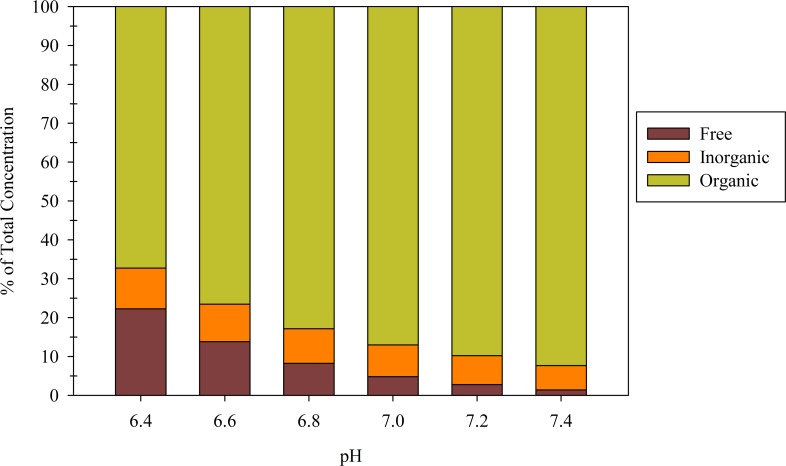
Results of Visual MINTEQ modeling showing the distribution of free, inorganic-complexed, and organic-complexed copper as a function of pH at DOC 1.5 mg/L and solid copper concentration of 10 mg/kg.

Copper was selected for additional analysis with chemical equilibrium modeling because it exceeded the reuse limits by such a large margin. The model output was used to assess the dissolved speciation between free, inorganic-complexed, and organic-complexed copper. Fig 1 shows dissolved copper speciation as a function of pH and that total dissolved copper decreases with increasing pH. Recall, all wash water samples fell within a pH range of 6.3–7.4. At the lower end of the measured pH range (pH 6.4), free copper is the dominant species (52%), but at pH 6.8, organic-complexed copper becomes the dominant species.

While Fig 1 shows that free and organic-complexed copper are dependent on pH, inorganic-complexed copper remained relatively constant. Recall that Tables 2 and 3 show large concentrations of copper and chloride. Despite the large concentrations, the two compounds have a stability constant of 0.43 [21]. This stability constant of <1 indicates that the two compounds have a low affinity for one another. Applying this weak interaction to Eq 2, copper is not likely to be bound to this inorganic ligand, even at higher chloride concentrations. The modeling results indicate carbonate was the dominant inorganic ligand.

To assess the impact of organics on speciation, Table 6 compares free, inorganic-complexed, and organic complexed copper concentrations as a function of pH and DOC concentration. Moving just 0.2 pH units, from 7.0 to 6.8, increases the free copper concentration two times (30 ppb vs. 62 ppb), indicating the significant impact of pH on potential compliance. The organic-complexed and inorganic-complexed copper concentrations also increased but not by nearly as much. Increasing the DOC input to 10 mg/L resulted in significant increases in organic-complexed copper but minimal impacts on free or inorganic-complexed copper (data not shown). The modeling results show that pH and DOC are both important constituents in copper speciation.

**Table 6:** Results of Visual MINTEQ modeling showing the comparison of total dissolved, free, inorganic-complexed, and organic-complexed copper as a function of pH at DOC 1.5 mg/L and solid copper concentration of 10 mg/kg.

pH	Total Dissolved	Free	Inorganic-Complexed	Organic-Complexed	Total Dissolved	Free	Inorganic-Complexed	Organic-Complexed
	Concentration (ppb)				%			
6.4	551	288	135	128	100	52	25	23
6.6	331	132	92	108	100	40	28	32
6.8	220	62	67	92	100	28	30	42
7.0	159	30	50	79	100	19	32	50
7.2	123	14	39	70	100	12	32	57
7.4	91	6	27	58	100	6	29	64

## Conclusions

A comprehensive sampling protocol that consisted of 16 heavy metal and 15 nonmetal constituents was used to characterize the wash water at three winter maintenance facilities. Nine of the 16 metals were detected in all samples, with copper, zinc, and iron having the highest concentrations. Comparison of raw wash water quality results to disposal and reuse limits showed that 4 of 19 of samples do not meet disposal limits, and 17 of 19 of samples do not meet reuse limits. Copper, oil and grease, and TSS exceed disposal limits. Copper, oil and grease, zinc, cyanide, and surfactants exceed reuse limits, and most samples are greater than 100% above the reuse limits, with copper exceeding by as much as 4,151%. Pre-treatment by an oil/water separator, clay-based filtration, and dilution were found to be inadequate to reduce concentrations to meet reuse limits.

Due to its strong presence in wash water, copper speciation was investigated with Visual MINTEQ. The results of the modeling indicated that increasing pH by only 0.2 units resulted in the free copper concentration decreasing from 288 ppb to 132 ppb. Increasing DOC concentrations also were shown to affect speciation. For this reason, DOC and pH are important parameters to consider when evaluating management options. Pretreatment is likely required, but simply monitoring pH and perhaps a surrogate for DOC (like UV 254 nm) could provide valuable insight into the likelihood of compliance prior to sending samples for metals testing. Use of the methods presented in this study provides the DOT with an approach to evaluate options to identify challenges associated with disposal and reuse of winter maintenance wash water.
